# Zoledronic acid induces apoptosis and S-phase arrest in mesothelioma through inhibiting Rab family proteins and topoisomerase II actions

**DOI:** 10.1038/cddis.2014.475

**Published:** 2014-11-13

**Authors:** S Okamoto, Y Jiang, K Kawamura, M Shingyoji, Y Tada, I Sekine, Y Takiguchi, K Tatsumi, H Kobayashi, H Shimada, K Hiroshima, M Tagawa

**Affiliations:** 1 Division of Pathology and Cell Therapy, Chiba Cancer Center Research Institute, Chiba, Japan; 2 Department of Respirology, Graduate School of Medicine, Chiba University, Chiba, Japan; 3 Department of Molecular Biology and Oncology, Graduate School of Medicine, Chiba University, Chiba, Japan; 4 Department of Thoracic Disease, Chiba Cancer Center, Chiba, Japan; 5 Department of Medical Oncology, Graduate School of Medicine, Chiba University, Chiba, Japan; 6 Department of Biochemistry, Graduate School of Pharmaceutical Sciences, Chiba University, Chiba, Japan; 7 Department of Surgery, School of Medicine, Toho University, Tokyo, Japan; 8 Department of Pathology, Tokyo Women's Medical University Yachiyo Medical Center, Chiba, Japan

## Abstract

Zoledronic acid (ZOL), a nitrogen-containing bisphosphonate, produced anti-tumor effects through apoptosis induction or S-phase arrest depending on human mesothelioma cells tested. An addition of isoprenoid, geranylgeraniol but not farnesol, negated these ZOL-induced effects, indicating that the ZOL-mediated effects were attributable to depletion of geranylgeranyl pyrophosphates which were substrates for prenylation processes of small guanine-nucleotide-binding regulatory proteins (small G proteins). ZOL-treated cells decreased a ratio of membrane to cytoplasmic fractions in RhoA, Cdc42 and Rab6 but less significantly Rac1 proteins, indicating that these proteins were possible targets for ZOL-induced actions. We further analyzed which small G proteins were responsible for the three ZOL-induced effects, caspase-mediated apoptosis, S-phase arrest and morphological changes, using inhibitors for respective small G proteins and siRNA for Cdc42. ZOL-induced apoptosis is due to insufficient prenylation of Rab proteins because an inhibitor of geranlygeranyl transferase II that was specific for Rab family proteins prenylation, but not others inhibitors, activated the same apoptotic pathways that ZOL did. ZOL suppressed an endogenous topoisomerase II activity, which was associated with apoptosis and S-phase arrest in respective cells because we detected the same cell cycle changes in etoposide-treated cells. Inhibitors for geranlygeranyl transferase I and for RhoA produced morphological changes and disrupted actin fiber structures, both of which were similar to those by ZOL treatments. These data demonstrated that anti-tumor effects by ZOL were attributable to inhibited functions of respective small G proteins and topoisomerase II activity, and suggested that cellular factors were involved in the differential cell cycle changes.

Bisphosphonates (BPs), synthetic analogues of pyrophosphates, are clinically in use for diseases with excessive bone absorption such as osteoporosis and malignancy-associated hypercalcemia. BPs administered *in vivo* are accumulated in the bone matrix and inhibit activities of osteoclasts.^1^ The first generation of BPs, without nitrogen in the structure, is converted into cytotoxic non-hydrolyzable ATP analogues and achieves cytotoxic effects thorough decreased mitochondrial membrane potentials.^2,3^ The second and the third generations, containing nitrogen, inhibit farnesyl pyrophosphate synthetase, a key enzyme in the mevalonate pathways, and deplete isoprenoid pools, which subsequently results in decreased prenylation of small guanine-nucleotide-binding regulatory proteins (small G proteins) (Supplementary Figure S1).^4^

Isoprenoid lipids, farnesyl pyrophosphate and geranylgeranyl pyrophosphate, are substrates for prenylation processes that mediate farnesylation and geranylgeranylation of small G proteins, respectively.^5,6^ Ras family proteins are either farnesylated by farnsyl transferase or geranylgeranylated by geranylgeranyl transferase I. In contrast, the majority of Rho family proteins and Rab family proteins are geranylgeranylated by geranylgeranyl transferase I and II, respectively. These lipid modifications are essential for most of small G proteins to bind to cytoplasmic and organelle membranes where prenylated small G proteins become functional, whereas unprenylated small G proteins remain in the cytoplasm and non-functional.^5^

The nitrogen-containing BPs (N-BPs) also induce cytotoxicity to osteoclasts, which is favorable for enhanced bone mineralization, and recent studies also showed that N-BPs had cytotoxic activities on tumors such as breast and prostate cancer.^7,8^ These cytotoxic actions are attributable to a number of mechanisms including apoptosis induction and anti-angiogenesis,^9,10^ but it is not well investigated as to which small G proteins produce the cytotoxic effects.

We recently showed that zoledronic acid (ZOL), which is one of the N-BPs to inhibit farnesyl pyrophosphate synthetase, produced cytotoxic activities to human mesothelioma.^11^ ZOL treatments induced apoptotic cell death or S-phase arrest in cell cycle, and moreover caused morphological changes from fibroblast-like to spherical shapes. In the present study, we examined what kinds of small G proteins are responsible to these ZOL-mediated effects using inhibitors or small interfering RNA (siRNA) for the respective small G proteins and for prenylating enzymes.

## Results

### ZOL induced apoptosis and S-phase arrest

**Figure 1:**
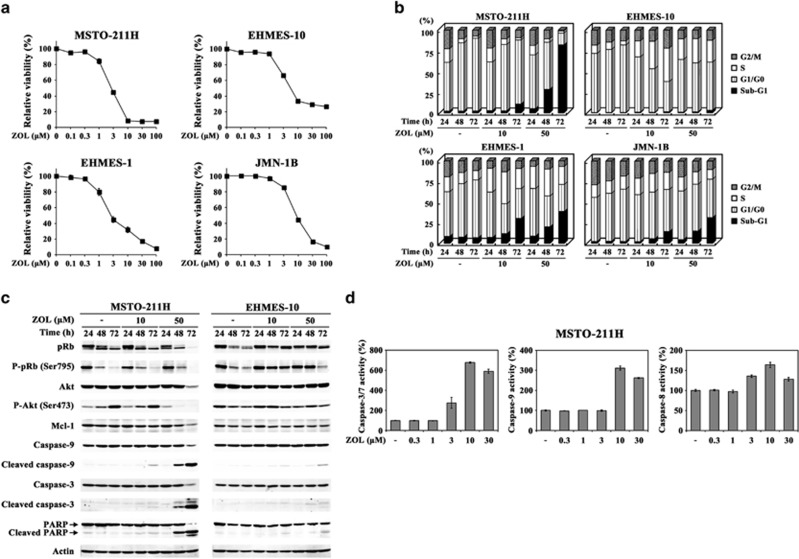

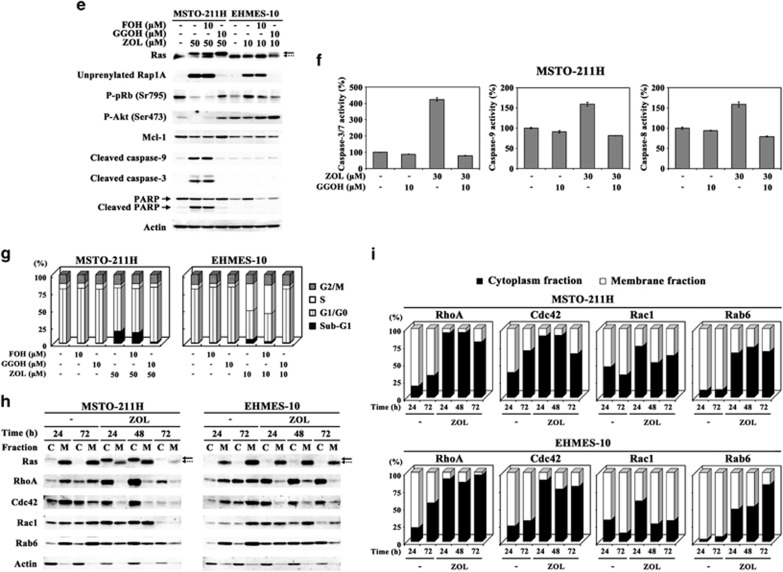
ZOL-mediated apoptosis and S-phase arrest through isoprenoid depletion. (**a**) Viability of cells treated with ZOL was measured with WST assay. The relative viability was calculated based on values of untreated cells as 100%. S.E. bars are shown (*n*=3). (**b** and **c**) Cells treated with different concentrations of ZOL were subjected to cell cycle (**b**) or western blot analyses with actin as the loading control (**c**). (**d** and **f**) Caspase activities in MSTO-211H cells treated with ZOL for 72 h (**d**) and with ZOL and/or GGOH for 48 h (**f**). The relative activity was expressed as a percentage of the untreated case. S.E. bars are shown (*n*=3). (**e** and **g**) Cells treated with agents as indicated for 48 h (MSTO-211H cells) or 72 h (EHMES-10 cells) and were subjected to western blot (**e**) or cell cycle analyses (**g**). Ras with high (arrow) and low (dotted arrow) molecular weights corresponds to unprenylated and prenylated forms, respectively. Actin is used as the control (**e**). (**h**) Lysates of cells treated with 50 *μ*M ZOL were separated into cytoplasm (c) or membrane (m) fraction and then probed with respective antibodies as indicated. (**i**) Differential ratios between cytoplasm and membrane fractions detected in western blot analyses (**h**). The intensity was determined with an imaging analyzer

We examined ZOL-mediated anti-tumor effects in human mesothelioma cells (Figure 1). Proliferation of four kinds of human mesothelioma cells was suppressed with ZOL treatments (Figure 1a). Cell cycle analyses demonstrated that ZOL increased sub-G1 fractions in MSTO-211H cells, S-phase populations in EHMES-10 cells, and both sub-G1 and S-phase populations in EHMES-1 and JMN-1B cells (Figure 1b). We therefore used MSTO-211H and EHMES-10 cells in further experiments as representative cells that showed increased sub-G1 and S-phase populations, respectively. We then examined signal pathways leading to cell death in MSTO-211H cells (Figure 1c). ZOL treatments decreased expression levels of Mcl-1 and phosphorylated Akt, but increased cleavages of caspase-9, -3 and poly (ADP-ribose) polymerase (PARP). In contrast, ZOL treatments minimally influenced these expression levels in EHMES-10 cells. We also showed that ZOL activated caspase-3, -7, -8 and -9 in MSTO-211H cells (Figure 1d). These data collectively indicated that ZOL induced apoptosis through caspase activations in MSTO-211H, whereas EHMES-10 cells were resistant to the apoptotic signals. ZOL-treated MSTO-211H cells showed dephosphorylation of pRb greater than untreated cells, but phosphorylated levels of pRb were maintained in ZOL-treated EHMES-10 cells compared with those of untreated cells.

### Geranylgeranyl pyrophosphate inhibited ZOL-mediated apoptosis and S-phase arrest

We investigated the involvement of farnesylation or geranylgeranylation in the ZOL-induced apoptosis and S-phase arrest. We examined an effect caused by addition of farnesol (FOH) and geranylgeraniol (GGOH), which were converted into farnesyl pyrophosphate and geranylgeranyl pyrophosphate, respectively, to determine ZOL's targets among small G proteins. A supplementary use of GGOH in MSTO-211H cells reduced ZOL-mediated downregulation of Mcl-1 expression, dephosphorylation levels of pRb and Akt, and cleavages of caspase-9, -3 and PARP (Figure 1e). Moreover, GGOH treatments inhibited ZOL-mediated activation of caspases (Figure 1f) and increase of sub-G1 fractions (Figure 1g). These data indicated that ZOL induced apoptosis through caspase activations. In contrast, FOH supplements did not influence these ZOL-mediated effects in MSTO-211H cells (Figures 1e and g). ZOL-induced S-phase arrest and phosphorylation of pRb in EHMES-10 cells were also inhibited with GGOH but not with FOH. These data demonstrated that ZOL-mediated apoptosis and S-phase arrest were attributed to reduced functions of geranylgeranylated but not farnesylated small G proteins. Western blot studies showed that ZOL-mediated unprenylation of Rap1A proteins was blocked with GGOH but not FOH administrations in both MSTO-211H and EHMES-10 cells (Figure 1e). On the other hand, the prenylation status of Ras proteins was differently modulated with ZOL and additional FOH or GGOH, and was not correlated with ZOL-mediated effects. Prenylation of Ras proteins was suppressed by ZOL and further inhibited by additional GGOH in MSTO-211H cells, whereas FOH rather augmented prenylation. The Ras prenylation in EHMES-10 cells remained unchanged with ZOL and with additional FOH treatments, but slightly inhibited by GGOH. These data collectively showed that Ras proteins were not responsible molecules in the ZOL-mediated effects.

### ZOL-induced cellular localization of small G proteins

We examined what kinds of small G proteins were unprenylated with ZOL treatments by testing the cellular distributions because some of antibodies specific to the unprenylated form was unavailable. Fractionated cytoplasmic and membrane portions were examined for the expressions of RhoA, Rac1 and Cdc42, belonging to the Rho family, and Rab6, one of the Rab family proteins (Figures 1h and i). ZOL treatments increased cytoplasmic distribution of RhoA, Cdc42, Rab6 and less significantly Rac1 in both of MSTO-211H and EHMES-10 cells. ZOL inhibited the translocation of Ras proteins from cytoplasm to membrane in MSTO-211H cells but did not influence the Ras distribution in EHMES-10 cells. These data indicated that ZOL suppressed membrane localization of small G proteins because of increased ungeranlygeranylated forms but the effects on Ras depended on cell types. Interestingly, ZOL-treated cells showed increase in total amounts of RhoA, Rac1 and less significantly Cdc42 but not Rab6, which were probably caused by a feedback mechanism in Rho family proteins production because of the decreased functional membrane-bound proteins (Supplementary Figure S2).

### Involvement of geranylgeranylated small G proteins in apoptosis or S-phase arrest

**Figure 2:**
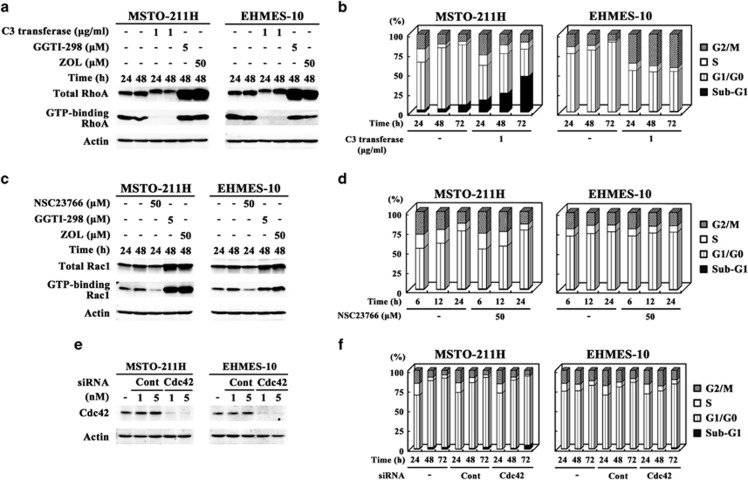
Influence of Rho inhibition on cell cycle. (**a**, **c** and **e**) Cells treated with an inhibitor as indicated, or transfected with Cdc42-siRNA or control-siRNA (Cont) were subjected to western blot analyses. (**b**, **d** and **f**) Cells were treated with C3 transferase (**b**) or NSC23766 (**d**), or transfected with 5 nM siRNA (**f**), and then were examined for cell cycle with flow cytometry

We used inhibitors or siRNA for respective small G proteins to identify possible small G proteins that were involved in ZOL-induced apoptosis or S-phase arrest (Figure 2). C3 transferase, an inhibitor of RhoA, did not influence an expression level of total RhoA in contrast to ZOL treatments, but decreased that of the GTP-binding form (Figure 2a). Cell cycle analyses showed that C3 transferase increased sub-G1 populations in MSTO-211H cells, and augmented G2/M-phase but not S-phase fractions in EHMES-10 cells (Figure 2b). NSC23766, an inhibitor of Rac1, which decreased the level of GTP-binding Rac1 without upregulating the whole protein level, did not influence on cell cycle in both MSTO-211H and EHMES-10 cells (Figures 2c and d). In contrast, ZOL treatments increased the whole and GTP-binding Rac1 as in the case of elevated whole RhoA expression. We used siRNA to downregulate Cdc42 protein expression because Cdc42 inhibitor was unavailable (Figure 2e). The siRNA for Cdc42 suppressed the protein expression, but did not influence cell cycle of MSTO-211H and EHMES-10 cells (Figure 2f). These data indicated that inhibition of RhoA but not Rac1 or Cdc42 functions induced apoptosis in MSTO-211H cells and none of the inhibition was associated with S-phase arrest in EHMES-10 cells.

**Figure 3:**
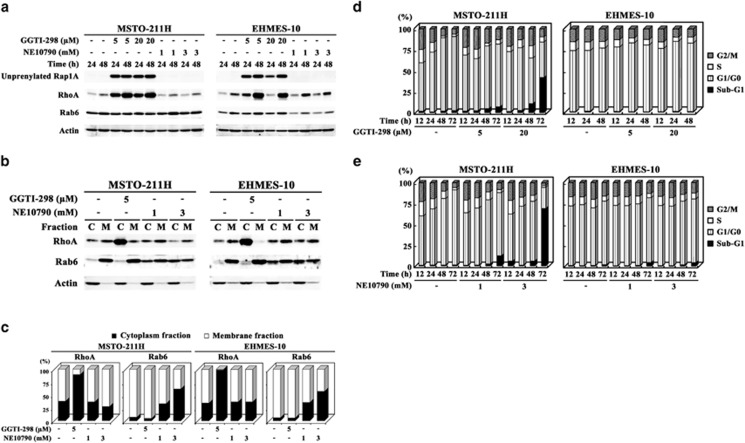
Influence of geranylgeranyl transferase I and II inhibition on cell cycle. (**a**) Cells treated with agents as indicated were subjected to western blot analyses. (**b**) Lysates of cells treated with agents for 48 h were separated into cytoplasm (c) or membrane (m) fraction and probed with respective antibodies. (**c**) Differential expression ratios between cytoplasm and membrane fractions in (**b**) were determined with an imaging analyzer. (**d** and **e**) Cells treated with agents as indicated were examined for cell cycle with flow cytometry

We further investigated the effects of inhibitors for geranylgeranylation, GGTI-298 and NE10790, which inhibit geranylgeranyl transferase I and II, respectively (Supplementary Figure S1). GGTI-298 treatments increased unprenylated Rap1A and whole RhoA levels as shown in ZOL-treated cells, but did not influence Rab6 expression (Figure 3a). The majority of RhoA in GGTI-298-treated cells was distributed in cytoplasmic fractions but that of Rab6 remained in membrane fractions (Figures 3b and c). In addition, GGTI-298 treatments upregulated Rac1 expression with enhanced GTP-binding Rac1 in MSTO-211H and less significantly in EHMES-10 cells (Figure 2c). NE10790 treatments did not influence the levels of unprenylated Rap1A or total RhoA expression, or fractionated RhoA distributions (Figures 3a–c). In contrast, NE10790-treated cells showed downregulation of Rab6 translocation to membrane in MSTO-211H and EHMES-10 cells although the expression levels were variable depending on the cells tested. We also examined cell cycle progression with these inhibitors. GGTI-298 increased sub-G1 populations in time- and dose-dependent manners in MSTO-211H cells but scarcely influenced the cell cycle in EHMES-10 cells (Figure 3d). NE10790 also augmented sub-G1 fractions in MSTO-211H cells but had little effects on cell cycle in EHMES-10 cells (Figure 3e). These data indicated that ungeranylgeranylation of Rho and/or Rab family proteins was responsible for ZOL-induced apoptosis but not for S-phase arrest.

### Apoptosis mediated by inhibited Rab functions and S-phase arrest by suppressed Topo II activity

**Figure 4:**
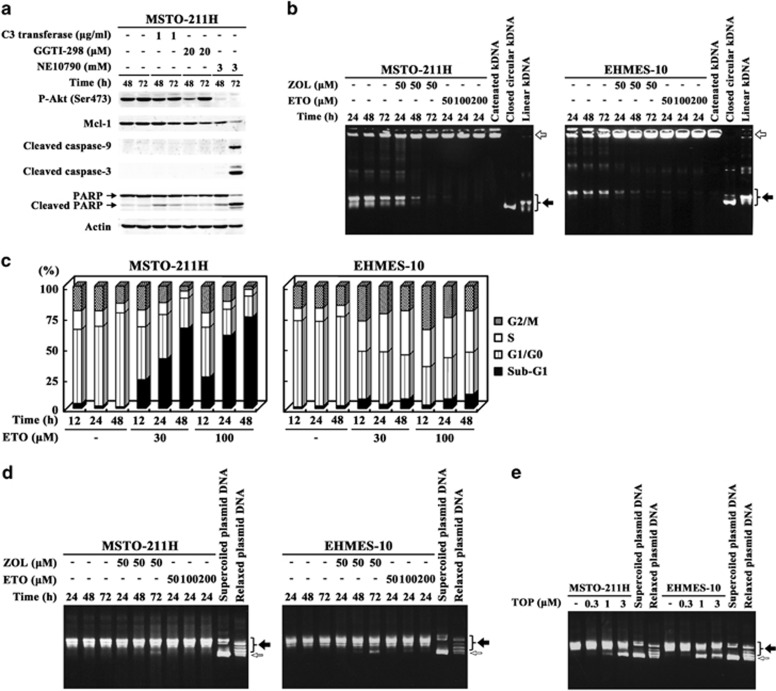
Involvement of geranylgeranylated small G proteins and topoisomerases in apoptosis and S-phase arrest. (**a**) Cells treated with agents as indicated were subjected to western blot analyses. (**b**) Cells treated with agents as indicated were assayed for the Topo II activity with catenated kinetoplast DNA with agarose gel electrophoresis. Open and closed arrows indicate catenated and decatenated kinetoplast DNA, respectively. ETO was used as the control. (**c**) ETO-treated cells were examined for cell cycle with flow cytometry. (**d** and **e**) Cells treated with agents as indicated were assayed for the Topo I activity with supercoiled plasmid DNA. Cells were treated with topotecan (TOP) for 24 h. Open and closed arrows indicate supercoiled and relaxed plasmid DNA, respectively

We investigated a possible mechanism of apoptosis induction by the inhibitors that increased sub-G1 fractions in MSTO-211H cells, C3 transferase, GGTI-298 and NE10790 (Figure 4a). C3 transferase-treated cells showed minimal cleavage of PARP without caspase-3 and -9 cleavages, and did not change Mcl-1 or phosphorylated Akt levels. GGTI-298-treated cells transiently downregulated the phosphorylation of Akt levels and minimally Mcl-1 levels, but did not induce cleavages of caspase-9, -3 or PARP. In contrast, NE10790 treatments decreased both phosphorylated Akt and Mcl-1 levels, and induced cleavages of caspase-9, -3 and PARP, all of which changes were the same as those observed in ZOL-treated MSTO-211H cells. These data collectively suggested that ZOL-induced apoptosis was attributable to the inhibition of Rab family proteins.

We investigated a possible mechanism of ZOL-induced S-phase arrest in EHMES-10 cells. We firstly examined topoisomerase (Topo) II activities in ZOL-treated cells (Figure 4b). Cell extracts of untreated MSTO-211H and EHMES-10 cells altered catenated kinetoplast DNA into decatenated form, demonstrating that the cells contained an endogenous Topo II activity. In contrast, extracts from ZOL-treated cells maintained catenated kinetoplast DNA form as shown in those from cells treated with etoposide (ETO), a Topo II inhibitor. We next analyzed cell cycle of ETO-treated MSTO-211H and EHMES-10 cells (Figure 4c). ETO treatments augmented sub-G1 fractions in MSTO-211H cells and upregulated S- and G2/M-phase populations in EHMES-10 cells. We further investigated whether ZOL inhibited Topo I activities with an assay to detect relaxation of supercoiled DNA (Figure 4d). Extracts of untreated MSTO-211H and EHMES-10 cells induced the relaxation of supercoiled DNA, demonstrating that they had an endogenous Topo I activity. ETO treatments also induced the relaxation because ETO was a specific Topo II inhibitor, and ZOL-treated cells had a minimal inhibitory activity to relax supercoiled DNA only at 72 h incubation. In contrast, cells treated with topotecan, a Topo I inhibitor, showed an inhibitory action on relaxing supercoiled DNA (Figure 4e). These data demonstrated that ZOL inhibited Topo II but scarcely Topo I activity and suggested that the blocking of Topo II activity was responsible for apoptosis in MSTO-211H cells and for S-phase arrest in EHMES-10 cells.

### ZOL-mediated morphological changes by inhibition of RhoA and Cdc42

**Figure 5:**
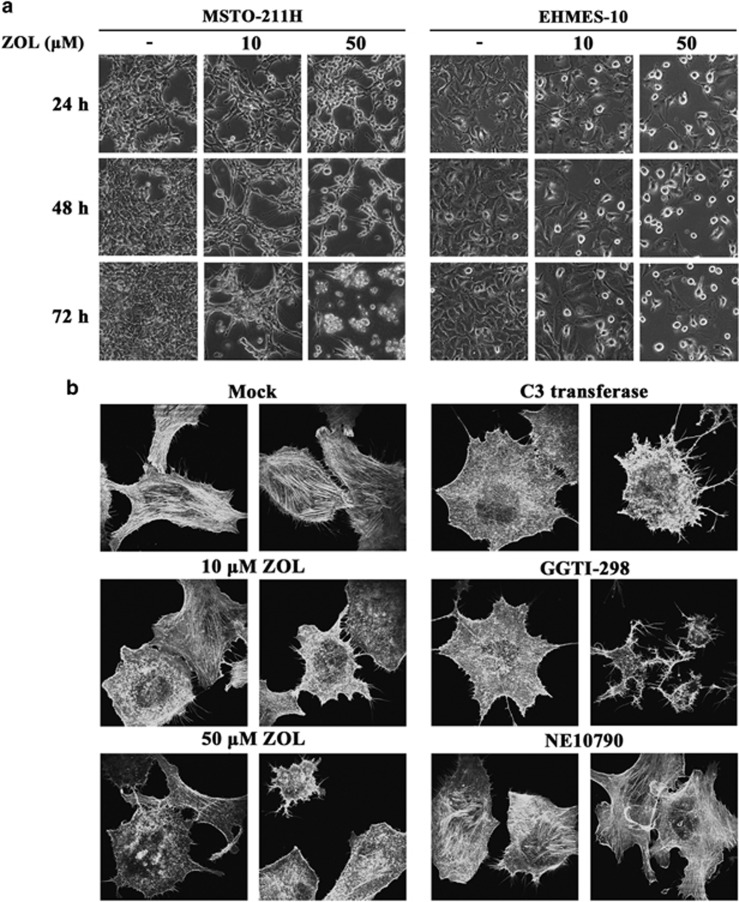
Morphological changes caused by ZOL and small G proteins inhibitors. (**a**) Microphotographs of ZOL-treated cells as indicated (magnification, × 200). (**b**) Structure of actin fibers in EHMES-10 cells which were treated with ZOL, 1 *μ*g/ml C3 transferase, 5 μM GGTI-298 or 1 mM NE10790 for 24 h and were stained with Alexa Fluor 488 phalloidin (magnification, × 630)

Cells treated with ZOL showed morphological changes, altering fibroblastic into round-shaped configurations and being less adhesive to a culture plate (Figure 5a). ZOL treatments caused the changes in time- and dose-dependent manners and the alterations were inhibited with GGOH but not FOH supplement (Supplementary Figure S3). We then examined which small G proteins were responsible for ZOL-mediated changes using the inhibitors. We focused on the round-shaped configuration in EHMES-10 as an indicator of ZOL-induced morphological changes because MSTO-211H cells treated with ZOL became easily detached from a culture plate and thereby were difficult to judge the change. GGTI-298-treated EHMES-10 cells showed almost the same alterations as shown in ZOL-treated cells, whereas NE10790-treated cells did not exhibit the shape changes (Supplementary Figure S4). These data indicated that ungeranylgeranylated Rab family proteins were irrelevant to the ZOL-mediated morphological changes. EHMES-10 cells treated with C3 transferase or Cdc42 siRNA but not with NSC23766 showed the round-shaped morphology although degree of the spheroid shape was lesser than that of GGTI-298-treated cells (Supplementary Figure S5). We also examined actin stress fiber structures as an indicator of the ZOL-induced cytoskeletal deformation (Figure 5b). Phalloidin-stained cells showed that ZOL treatments suppressed the actin stress fiber formation, and that C3 transferase-treated and GGTI-298-treated cells exhibited similar fiber-disrupted patterns. In contrast, NE10790 treatments did not influence the actin stress fiber conformations. These data suggested that morphological changes induced by ZOL treatments were due to inhibition of RhoA and Cdc42.

## Discussion

In the present study, we investigated possible molecules responsible for ZOL-induced apoptosis, S-phase arrest and morphological changes in mesothelioma. We examined the target molecules with supplementary isoprenoids, specific inhibitors and siRNA for small G proteins because ZOL-mediated unprenylation of small G proteins resulted in the loss of functions. These experiments collectively indicated that the ZOL-mediated apoptosis and morphological changes were due to inhibition of Rab family and Rho family proteins, respectively. The S-phase arrest observed in ZOL-treated cells was not attributed to unprenylation of small G proteins but to inhibition of Topo II activity.

N-BPs can produce cytotoxic effects, but the precise mechanism remains uncharacterized in particular as for which small G proteins are involved. We examined differential effects of FOH and GGOH and demonstrated that geranylgeranylation was crucial for the cytotoxicity. Treatments with GGTI-298 or NE10790 confirmed that deficient geranylgeranylation induced cell death and we further demonstrated that C3 transferase but not NSC23766 or siRNA for Cdc42 induced cell death. Expression patterns of apoptosis-related molecules indicated that NE10790 but not GGTI-298 or C3 transferase activated the same apoptotic pathways that ZOL did. We thereby concluded that ZOL-induced apoptotic cell death was attributable to the loss of Rab family proteins' functions. A previous study also demonstrated that NE10790 induced apoptosis without cell cycle arrest but did not investigate precise mechanisms as to which apoptosis signals were involved.^12^ We did not determine in the present study what kinds of Rab family proteins contributed to ZOL-induced apoptosis, but the experiments with NE10790 narrowed down the targeted Rab family proteins. Rab family proteins possess a one- or two-cysteine-containing motif at the C terminal, and NE10790 inhibits geranylgeranylation of only the second of the two-cysteines residues.^13, 14, 15^ The targets of NE10790 are consequently Rab family proteins with two-cysteine-containing motifs such as Rab1, Rab5 and Rab6. Some Rab family proteins are highly expressed in tumors and can be involved in progression and invasion of the tumors.^16,17^ Rab family proteins are also involved in intracellular transport among organelles, which is crucial for cell survival. We thereby presume that the Rab functions in tumors cannot be substituted by other small G proteins.

Production of a cytotoxic ATP analogue is another mechanism of ZOL-mediated cytotoxicity.^18^ ZOL-induced depletion of isoprenoid can stimulate a feedback mechanism in the mevalonate pathway and enhance the hydroxymethylglutaryl-CoA reductase activity accordingly (Supplementary Figure S1). The upregulated enzyme activity promotes accumulation of isopentenyl pyrophosphate that is subsequently metabolized to the ATP analogue, ApppI, which induces the mitochondria-mediated apoptosis.^18,19^ The first generation of non-N-BPs, which does not influence prenylation processes of small G proteins, was converted into an ATP analogue similar to ApppI in the structure and achieved apoptosis by reducing mitochondria membrane potentials.^3,20^ The present study, however, did not show that ApppI was responsible for ZOL-mediated apoptosis because ApppI was currently unavailable. Supposing that geranylgeranyl pyrophosphate is an end product and stimulates the feedback mechanism, the present results that GGOH but not FOH cancelled ZOL-mediated effects are consistent with the assumption that ApppI played a role in ZOL-mediated effects. In contrast to the present study, a recent study showed that FOH as well as GGOH supplements suppressed ZOL-mediated apoptosis by decreasing ApppI amounts in non-mesothelioma cells.^21^ These data hence collectively suggest that production of ApppI can be a possible mechanism of ZOL-mediated effects although the feedback mechanism and a role of ApppI in the apoptosis are subjected to cell and/or tissue type difference.

The present study indicated that none of the inhibitors for geranylgeranylation of small G proteins contributed to S-phase arrest and showed that the majority of geranylgeranylated small G proteins were irrelevant to increase in S-phase populations. We then examined the possibility that an ATP analogue was responsible for S-phase arrest by inhibiting Topo II activity in mesothelioma. We demonstrated that ZOL specifically suppressed Topo II actions, which is probably mediated by the ATP analogue because Topo II but not Topo I requires ATP for the catalytic activity.^22, 23, 24^ ETO-treated MSTO-211H and EHMES-10 cells showed apoptosis and S-phase arrest, respectively, as found in ZOL-treated cells. We further investigated whether clodronate, which belongs to the first generation of BPs and produces an ATP analogue, AppCCl_2_p, similar to ApppI in the structure, induced S-phase arrest in EHMES-10 cells (Supplementary Figure S6). Clodronate treatments reduced cell viability (Supplementary Figure S6a) and augmented sub-G1 populations without S-phase arrest at high concentration in MSTO-211H and EHMES-10 cells (Supplementary Figures S6b and c). Previous studies also showed that clodronate-treated non-mesothelioma cells induced apoptosis but not S-phase arrest.^25^ We thereby presume that ApppI has a different affinity to Topo II from AppCCl_2_p, and a previous study in fact demonstrated different susceptibility of osteoclasts to ApppI and AppCCl_2_p.^18^ ZOL treatments stimulate ApppI accumulations but further studies are required to confirm that ApppI suppresses Topo II activity and induces cell cycle changes.

ZOL treatments induced insufficient adhesion to plates in MSTO-211H cells and round-shape configurations in EHMES-10 cells. GGOH supplements reduced these morphological changes, demonstrating that ungeranylgeranylation of small G proteins contributed to the actions. We showed that GGTI-298 and C3 transferase disturbed formation of actin stress fibers and that siRNA-Cdc42 induced round-shaped configurations in EHMES-10 cells. The GGTI-298-induced alterations were similar to those by ZOL but NE10790 or NSC23766 had no effect on the morphological changes. These data suggest that ZOL-produced changes were resulted from ungeranylgeranylation of Rho family proteins excluding Rac1. We further investigated whether the morphological changes were linked with S-phase arrest because alterations in cell shapes can influence cell growth.^26^ NCI-H28 cells, a human mesothelioma cell line, treated with ZOL showed increased S-phase fractions as observed in EHMES-10 cells but did not induce any morphological changes (Supplementary Figure S7). These data implied that induction of S-phase arrest was irrelevant to ZOL-produced morphological changes.

In conclusion, we investigated candidate target molecules of ZOL with inhibitors or siRNA for small G proteins and for topoisomerase enzymes, and found that ZOL-induced effects in mesothelioma cells were associated with functional suppression of respective small G proteins and with inhibition of Topo II activities. Apoptosis was linked with inhibition of Rab family functions and Topo II actions, and S-phase arrest was associated with suppressed Topo II activities. Morphological changes with disrupted actin stress fiber structures were caused by inhibited functions of Rho family protein. Nevertheless, these pharmacological actions were influenced by cell type difference. The present data suggest suitable target molecules for cancer therapy in future and also imply a possible combinatory use of different types of anti-cancer agents for mesothelioma treatments.

## Materials and Methods

### Cells and reagents

Human mesothelioma, MSTO-211H and NCI-H28 cells, were purchased from American Type Culture Collection (Manassas, VA, USA), and EHMES-1, EHMES-10 and JMN-1B cells were provided by Dr Hamada (Ehime University, Ehime, Japan). ZOL were purchased from Novartis (Basel, Switzerland), and FOH, GGOH, GGTI-298, ETO, topotecan and clodronate were from Sigma-Aldrich (St Louis, MO, USA). C3 transferase and NSC23766 were from Cytoskeleton (Denver, CO, USA) and Merck Millipore (Billerica, MA, USA), respectively. NE10790 were provided by Dr Ebetino FH (Warner Chilcott, Dundalk, Ireland). We purchased siRNA duplex targeting Cdc42 and non-specific control siRNA from Invitrogen (Carlsbad, CA, USA). Transfection of cells with the siRNA was conducted with Lipofectamine RNAiMAX (Invitrogen).

### Cell cycle analysis

Cells were fixed in 100% ethanol, treated with RNase (50 *μ*g/ml) and stained with propidium iodide (50 *μ*g/ml). The fluorescence intensity was analyzed with FACSCalibur and CellQuest software (BD Biosciences, San Jose, CA, USA).

### Cell proliferation activity

Cell growth was examined with a cell-counting WST kit (Dojindo, Kumamoto, Japan) (WST assay). The amount of formazan produced was determined with the absorbance at 450 nm and the relative viability was calculated based on the absorbance without any treatments.

### Western blot analysis

Cells lysates were subjected to sodium dodecyl sulfate polyacrylamide gel electrophoresis and the proteins were transferred to a nitrocellulose membrane. The membrane was hybridized with antibodies against pRb, phosphorylated pRb at Ser 795, Akt, phosphorylated Akt at Ser 473, caspase-3, cleaved caspase-3, caspase-9, cleaved caspase-9, PARP (Cell Signaling, Danvers, MA, USA), Mcl-1, unprenylated Rap1A, Rab6 (Santa Cruz Biotech, Santa Cruz, CA, USA), RhoA, Cdc42, Rac1 (Cytoskeleton), Ras (BD Biosciences) or actin (Sigma-Aldrich). The membranes were developed with the ECL system (GE Healthcare, Buckinghamshire, UK). Membrane and cytoplasm fractions were separated with a native membrane extraction kit (Merck Millipore) according to the manufacturer's protocol. Intensity of hybridized bands was determined with the public domain Image J program (available at http://rsbweb.nih.gov/ij). GTP-binding RhoA and Rac1 proteins were isolated with a pull-down assay using a Rho activation assay biochem kit and a Rac1 activation assay biochem kit (Cytoskeleton) according to the manufacturer's protocol.

### Caspase activity

Cells were examined for the caspase-3/7, -8 and -9 activities with respective Caspase-Glo kits according to the manufacturer's protocol (Promega, Madison, WI, USA). The relative activity was calculated based on luminescence intensity of cells without any treatment.

### Topoisomerase activity

Topo I and Topo II activities were determined with corresponding kits according to the manufacturer's protocol (TopoGEN, Port Orange, FL, USA). Cell extract and substrate DNA, supercoiled DNA for Topo I or catenated kinetoplast DNA for Topo II, were incubated with (for Topo II activity) and without ATP (Topo I). The reaction mixtures were digested with proteinase K and then subjected to agarose gel electrophoresis.

### Immunofluorecence analysis

Cells cultured on a Matrigel (BD biosciences)-coated cover glass were fixed with 3.7% formaldehyde and treated with Alexa Fluor 488 phalloidin (Invitrogen) followed by ProLong Gold antifade reagent (Invitrogen). The fluorescence images were taken with a confocal microscope, Leica TCS-SPE (Leica microsystems, Tokyo, Japan).

## Abbreviations

BPs: bisphosphonates
ETO: etoposide
FOH: farnesol
GGOH: geranylgeraniol
N-BPs: nitrogen-containing bisphosphonates
PARP: poly (ADP-ribose) polymerase
siRNA: small interfering RNA
small G proteins: small guanine-nucleotide-binding regulatory proteins
Topo: topoisomerase
ZOL: zoledronic acid
