# Supplementary material for: Zoledronic acid induces apoptosis and S-phase arrest in mesothelioma through inhibiting Rab family proteins and topoisomerase II actions
Source: Cell Death Dis. 2014 Nov 13;5(11):e1517–. doi: 10.1038/cddis.2014.475 (PMC4260733; doi:10.1038/cddis.2014.475)
Supplement: Supplementary Information [file cddis2014475x1.doc]

**SUPPLEMENTARY FIGURE LEGENDS**

**Supplementary Figure S1. Molecules involved in the mevalonate pathway and prenylation of small G proteins.** The agents used in the present studies are also shown.

**Supplementary Figure S2. ZOL enhanced expression levels of small G proteins.** Cells treated with ZOL as indicated were subjected to Western blot analyses.

**Supplementary Figure S3. Morphological changes mediated by ZOL was inhibited by GGOH.** MSTO-211H cells were treated with 10 μM FOH or GGOH and 50 μM ZOL for 48 h. EHMES-10 cells were treated with 10 μM FOH or GGOH and 10 μM ZOL for 72 h. These cell morphologies were shown in microscopic photos (magnification, × 200).

**Supplementary Figure S4. Influence of GGTase I and II inhibitors on cell morphology.** (**a** and **b**) Microphotographs of EHMES-10 cells treated with GGTI-298 (**a**) or NE10790 (**b**) (magnification, × 200).

**Supplementary Figure S5. Influence of small G protein inhibitors and Cdc42-siRNA on cell morphology.** (**a** and **b**) Microphotographs of EHMES-10 cells treated with 1 μg/ml C3 transferase or 50 μM NSC23766 (**a**), or transfected with 3 nM siRNA as indicated (**b**) (magnification, × 200).

**Supplementary Figure S6. Cytotoxic effects induced by CLO.** (**a**) Cells were treated with CLO for 3 days and the viabilities were measured with WST assay. The relative viability was calculated based on values of untreated cells as 100%. Means of triplicated samples and SE bars are shown. (**b** and **c**) Cells treated with CLO were examined for cell cycle with flow cytometry.

**Supplementary Figure S7. Influence of ZOL on cell cycle and morphological changes.** (**a**) NCI-H28 cells treated with ZOL were examined for cell cycle with flow cytometry. (**b**) Microphotographs of NCI-H28 cells untreated or treated with ZOL (magnification, × 200).
