# Supplementary figures and images for: Zoledronic acid induces apoptosis and S-phase arrest in mesothelioma through inhibiting Rab family proteins and topoisomerase II actions
Source: Cell Death Dis. 2014 Nov 13;5(11):e1517–. doi: 10.1038/cddis.2014.475 (PMC4260733; doi:10.1038/cddis.2014.475)

## Supplementary Figure S1

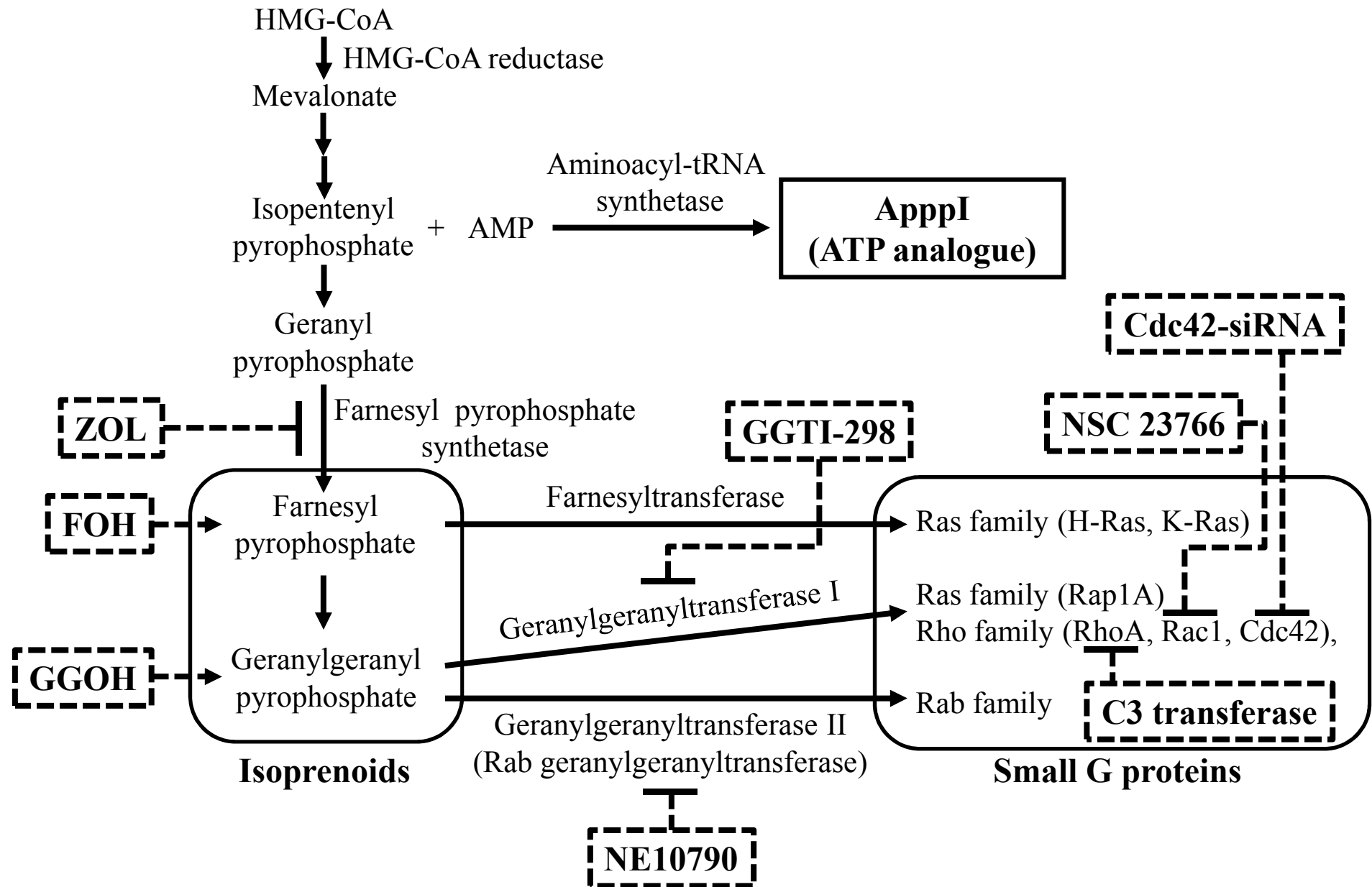

Supplement: Supplementary Figure S1 [file cddis2014475x2.pdf]

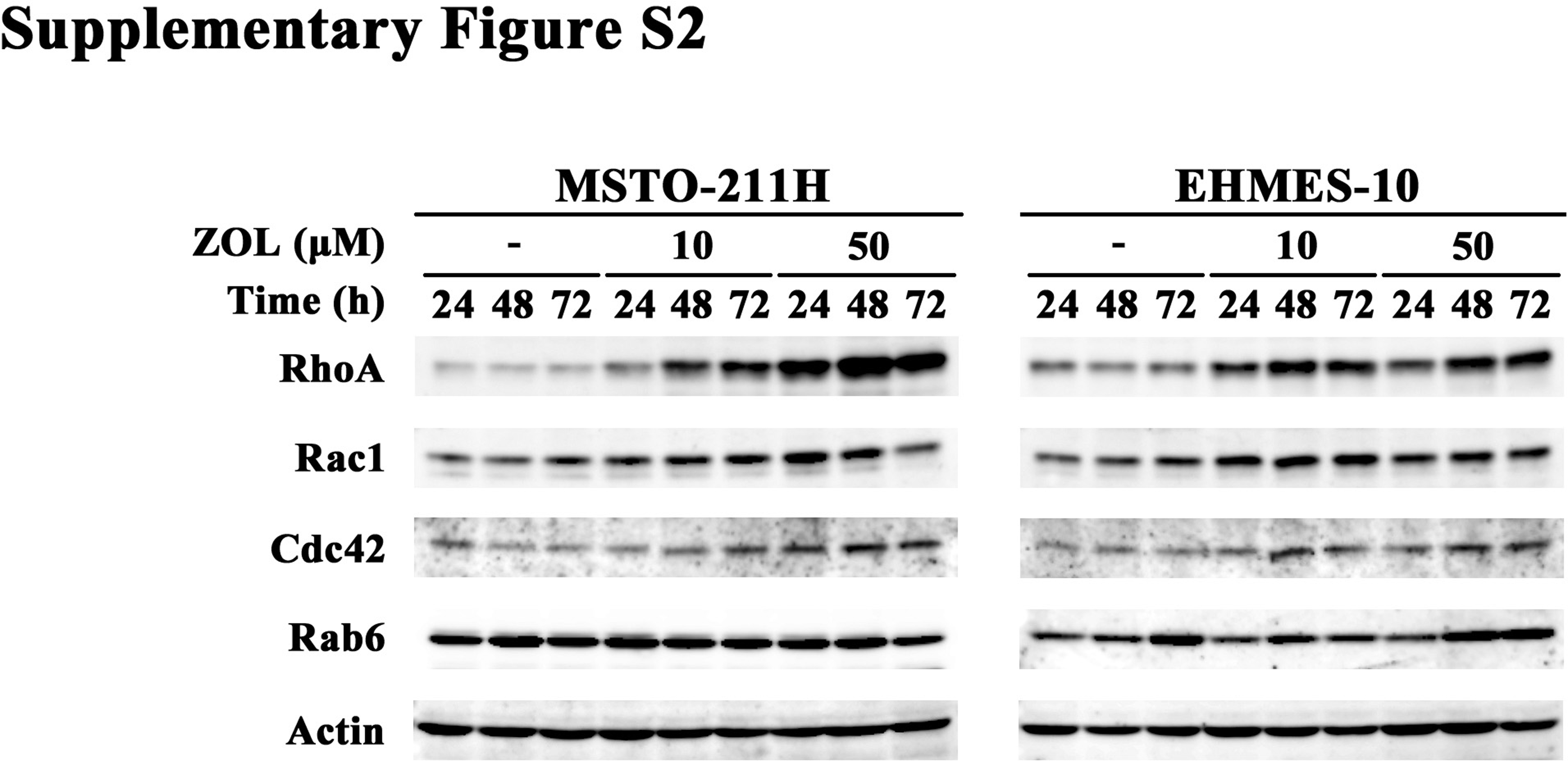

Supplement: Supplementary Figure S2 [file cddis2014475x3.tif]

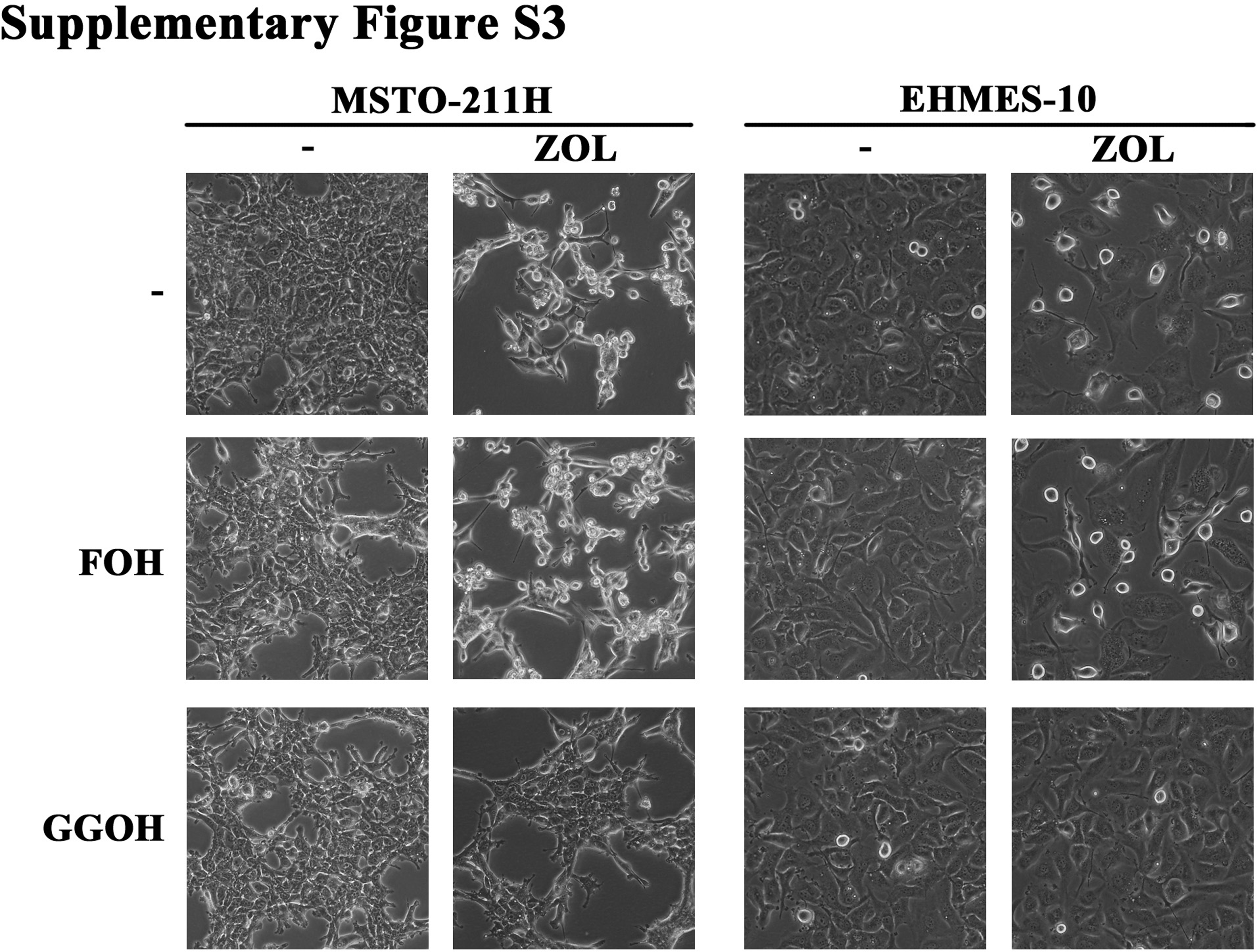

Supplement: Supplementary Figure S3 [file cddis2014475x4.tif]

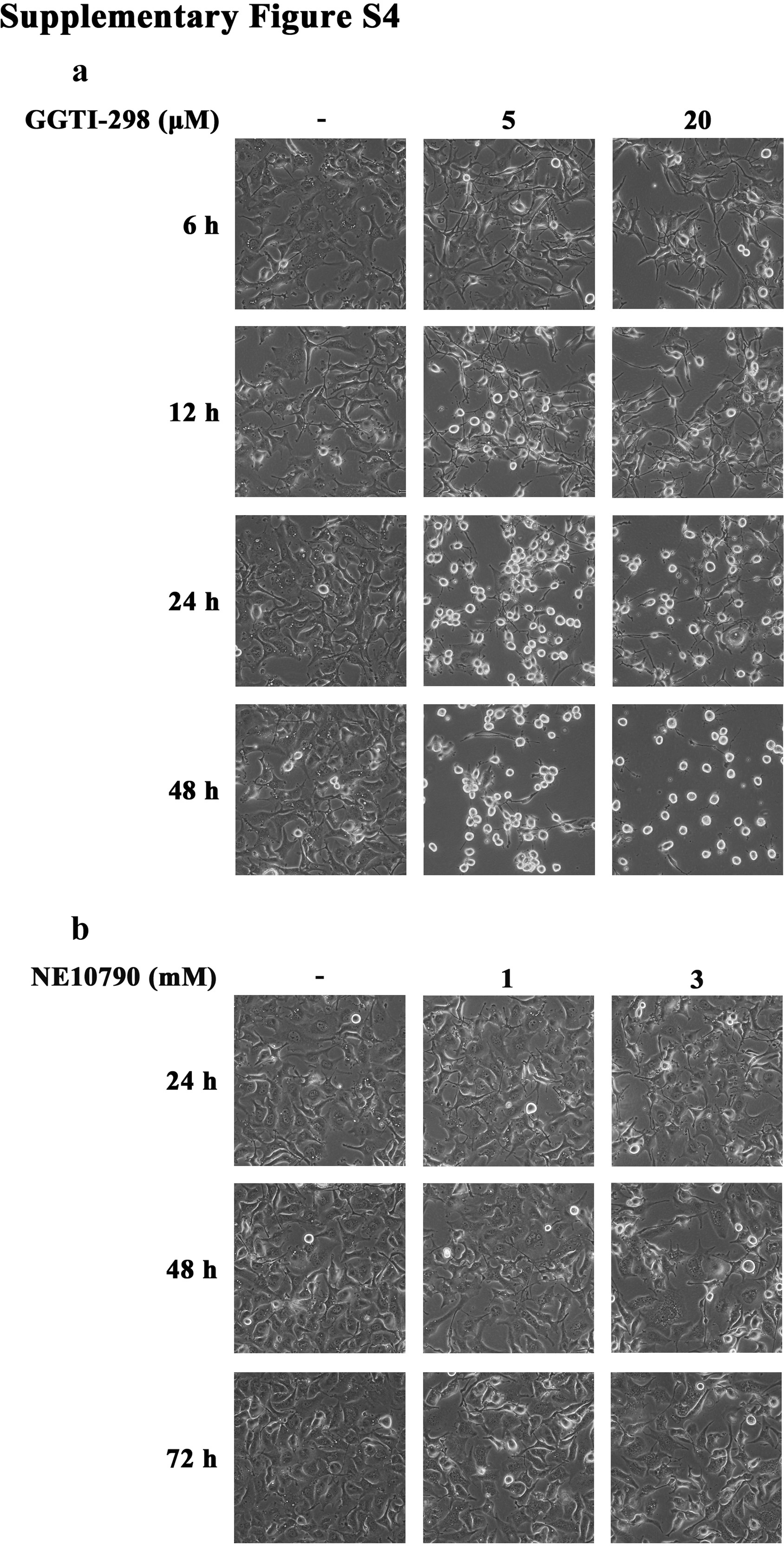

Supplement: Supplementary Figure S4 [file cddis2014475x5.tif]

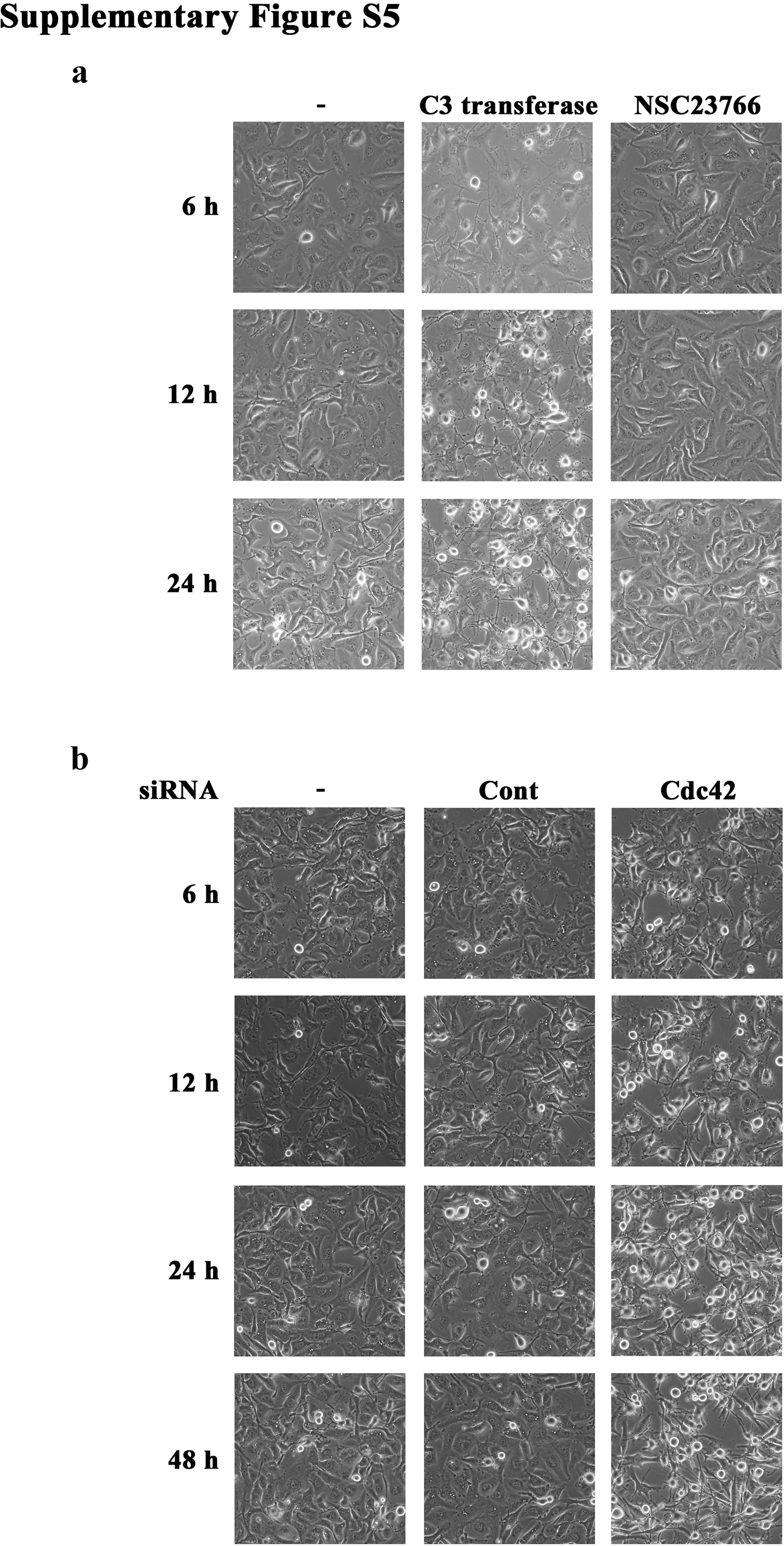

Supplement: Supplementary Figure S5 [file cddis2014475x6.tif]

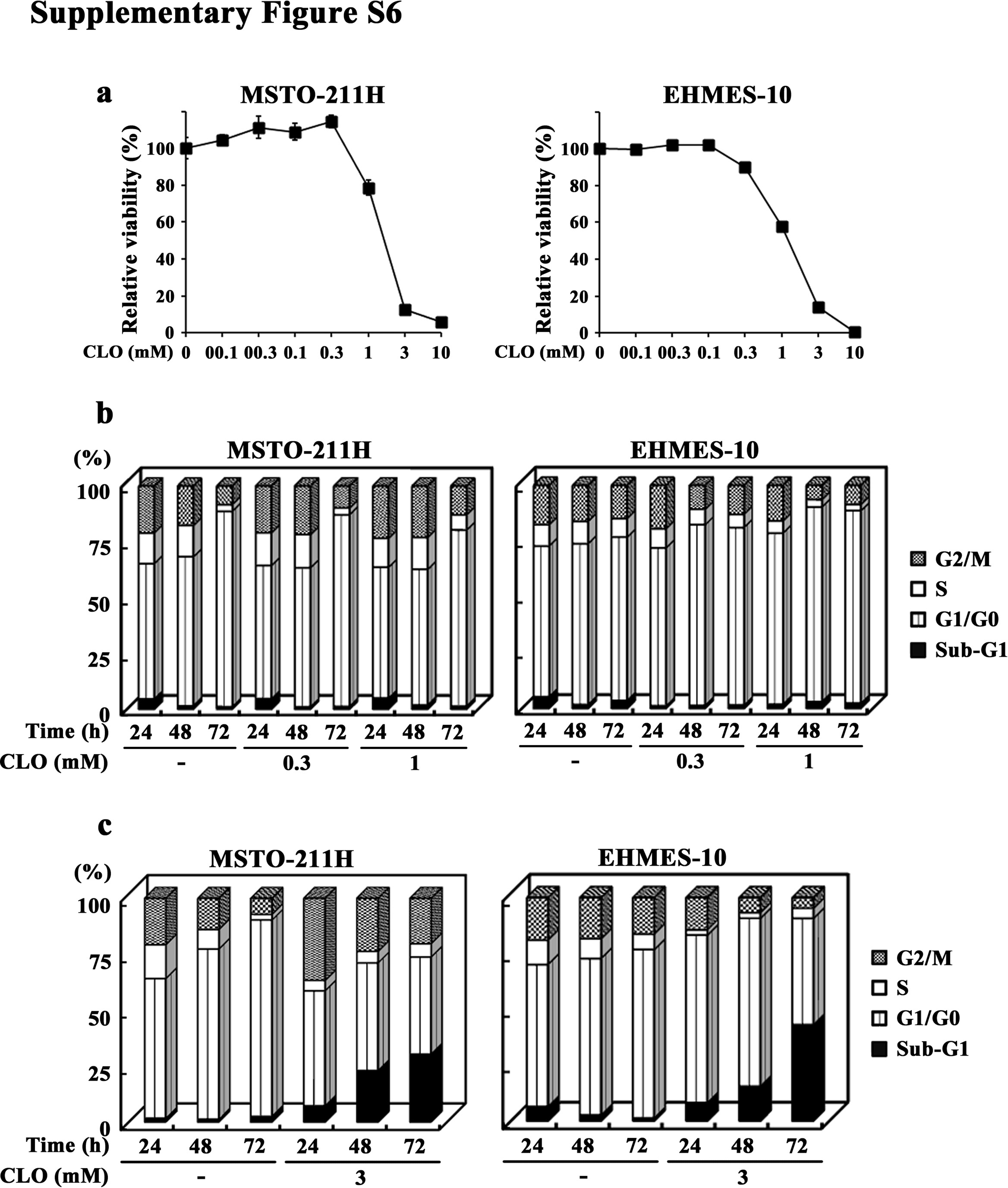

Supplement: Supplementary Figure S6 [file cddis2014475x7.tif]

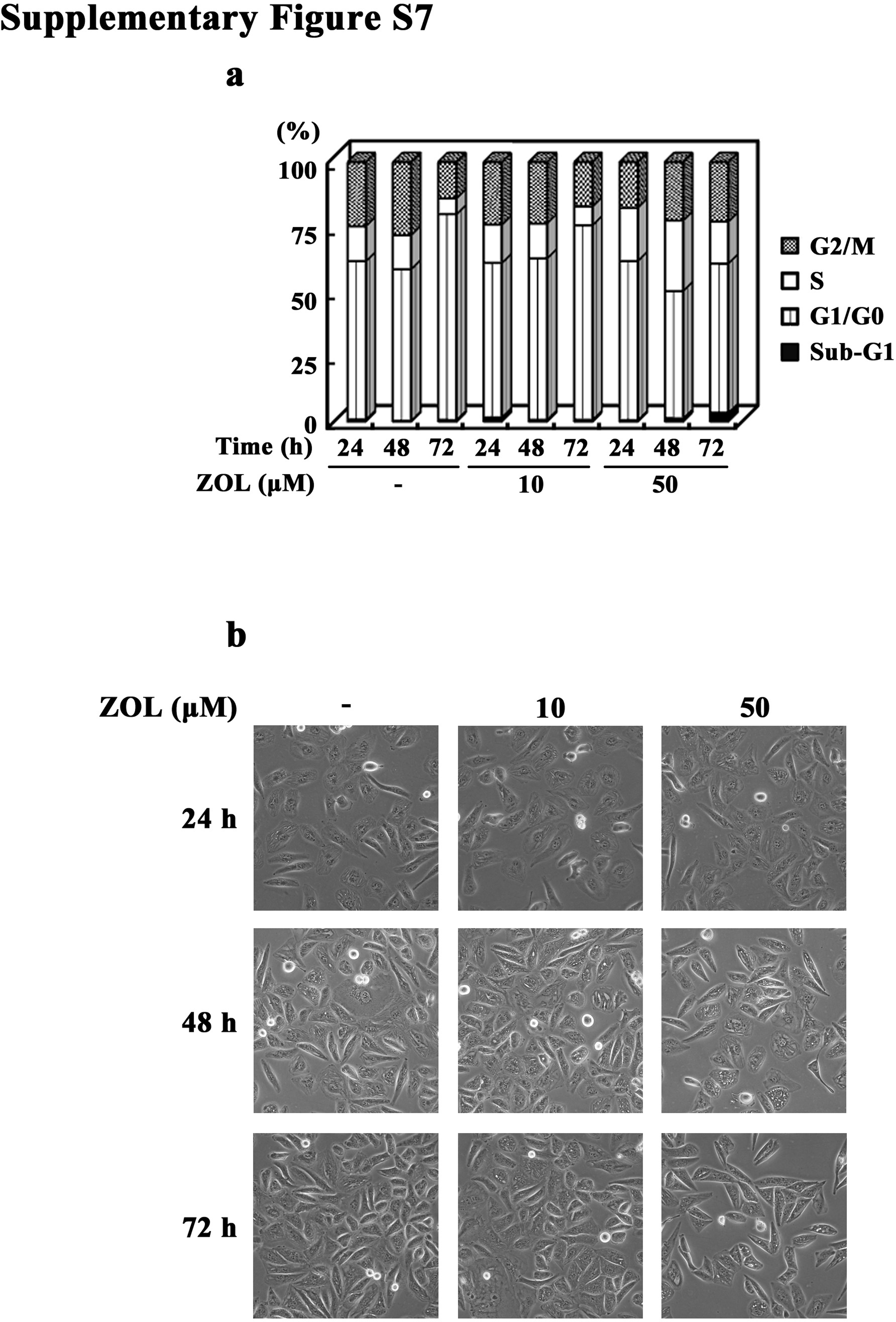

Supplement: Supplementary Figure S7 [file cddis2014475x8.tif]
